# Neurobiological Links Between Endodontic Pain and Temporomandibular Disorders: A Comprehensive Review

**DOI:** 10.7759/cureus.115695

**Published:** 2026-09-03

**Authors:** Akshay Katara, Saurav Bathla, Suyash Pratap Singh, Richa Singh, Mohit Galani, Navin Kumar D, Ashish Kumar Shukla, Ritik Kashwani

**Affiliations:** 1 Department of Dentistry, Omni Family Health, Bakersfield, USA; 2 Department of Conservative Dentistry and Endodontics, Teerthanker Mahaveer Dental College and Research Centre, Teerthanker Mahaveer University, Moradabad, IND; 3 Department of Conservative Dentistry and Endodontics, Institute of Dental Sciences, Bareilly International University, Bareilly, IND; 4 Department of Oral and Maxillofacial Surgery, Shree Bankey Bihari Dental College & Research Centre, Ghaziabad, IND; 5 Department of Dentistry, All India Institute of Medical Sciences, Rajkot, IND; 6 Department of Periodontology, Madha Dental College &amp; Hospital, Tamil Nadu Dr. M.G.R. Medical University, Chennai, IND; 7 Department of Radiodiagnosis, Santosh Medical College and Hospital, Santosh Deemed to be University, Ghaziabad, IND; 8 Oral Medicine and Radiology, School of Dental Sciences, Sharda University, Greater Noida, IND

**Keywords:** central sensitization, endodontic pain, neuroplasticity, nociception, orofacial pain, peripheral sensitization, persistent dentoalveolar pain, post-traumatic trigeminal neuropathy, temporomandibular disorders, trigeminal nerve

## Abstract

Endodontic (pulpal and periapical) pain and temporomandibular disorders (TMDs) are often considered to be clinical entities in different dental subspecialties. Both are present in the same sensory system (the trigeminal nerve and its central projections); however, there is recent evidence to suggest that some common neurobiological substrates exist between the two. Mechanistic connections between the inflamed/instrumented tooth and the masticatory muscle and joint pain associated with TMD consist of peripheral sensitization at the inflamed/instrumented tooth, activity-dependent changes in trigeminal ganglion neurons, and activity-dependent changes in the trigemino-thalamo-cortical axis. The current review is an overview of recent literature (2020-2026) that underscores the common neuroanatomy, transition of acute nociceptive endodontic pain to sensitized and even neuropathic states, and clinical overlap in these conditions for persistent dentoalveolar pain and post-traumatic trigeminal neuropathy. This knowledge is directly relevant to diagnosis, to avoiding iatrogenic roll-down, and to management strategies that intervene in sensitised central pathways and not just in the tooth. We believe that an understanding of endodontic pain and TMD should be considered a continuum of trigeminal nociceptive and neuroplastic processes.

## Introduction and background

Patients commonly seek orofacial care for pain resulting from dental causes and pain associated with the temporomandibular joint (TMJ) [1]. Patients frequently describe endodontic pain as some of the most severe pain that they have ever had, caused by the inflammation of the pulpal tissues, periapical disease, or the result of root canal treatment [2]. By contrast, TMDs are a group of musculoskeletal problems affecting the TMJ, masticatory muscles, and associated structures that can be intermittent, diffuse, and hard to locate [3]. In normal practice, these presentations are treated separately, with the endodontist treating the tooth and the orofacial pain specialist treating the joint and muscles [4].

This is a pragmatic division, yet there is an important biological reality that is lost. These structures are all connected to the trigeminal nerve, and they all send nociceptive signals to common second-order neurons in the brainstem, which, in turn, are along common thalamocortical pathways [5]. Peripheral input in one territory can then affect processing of input in another, and a strong enough or prolonged peripheral insult can induce enduring alterations of central pain networks [6]. This overlap is increasingly recognized in classification systems, which regulate this field and are now placing odontogenic pain, TMD, post-traumatic trigeminal neuropathic pain, and persistent idiopathic dentoalveolar pain in a single organising structure of orofacial pain [7].

The trigeminal neuroanatomy shared between both systems, the mechanisms of peripheral and central sensitization, the imaging and molecular changes associated with neuroplasticity, and the potential pathways that lead to an endodontic insult seeding persistent or neuropathic pain that clinically resembles TMD are discussed [8]. Lastly, we examine the clinical and therapeutic significance of these disorders as different manifestations of a dysfunction of the trigeminal system. The aim of this review is to discuss the neurobiological links between endodontic pain and TMD based on recent evidence.

## Review

This narrative review was undertaken to synthesize recent evidence on the neurobiological links between endodontic pain and temporomandibular disorders (TMDs), with particular emphasis on trigeminal neuroanatomy, peripheral and central sensitization, neuroplasticity, post-traumatic trigeminal neuropathy (PTTN), diagnostic overlap, and therapeutic implications. The literature search focused primarily on publications from January 2020 through August 2026. PubMed/MEDLINE, Scopus, Web of Science, and Google Scholar were searched using combinations of the following terms: “endodontic pain”, “pulpal pain”, “periapical pain”, “post-endodontic pain”, “temporomandibular disorders”, “TMD”, “trigeminal nerve”, “trigeminal neuropathy”, “post-traumatic trigeminal neuropathy”, “central sensitization”, “peripheral sensitization”, “neuroplasticity,” “orofacial pain”, “neuroimaging”, and “neurophysiological assessment”. Boolean operators (AND/OR) were used to combine terms where appropriate. Reference lists of relevant review articles and key primary studies were also examined manually to identify additional publications relevant to the scope of the review.

Studies were considered for inclusion when they addressed one or more of the following themes: (1) neurobiological mechanisms underlying endodontic or dentoalveolar pain; (2) trigeminal peripheral or central sensitization; (3) TMD-related neuroplasticity or neuroimaging findings; (4) PTTN or persistent pain following dental or endodontic procedures; (5) diagnostic overlap between dental pain, TMD, and other orofacial pain disorders; or (6) neurophysiological or therapeutic approaches relevant to these conditions. Peer-reviewed original research articles, systematic reviews, narrative or literature reviews, and clinically relevant evidence published in English were considered. Studies unrelated to trigeminal/orofacial pain mechanisms, studies focused exclusively on non-orofacial pain conditions, and publications without sufficient relevance to the review objectives were excluded.

Shared neuroanatomy: the trigeminal system as a common substrate

The trigeminal nerve, located in the trigeminal ganglion [9], has two divisions: the maxillary division (V2) and the mandibular division (V3), supplying the teeth, periodontium, TMJ, and masticatory muscles with sensory innervation. The density and vulnerability of this system of nerves in the pathway between the brain and the face are highlighted by anatomical studies, which continue to clarify the pathways of the trigeminal system and the bony and dural relationships in which these nerves pass [10]. Primary afferents from pulpal, periradicular, muscular, and articular tissue project centrally to the spinal trigeminal nucleus, especially the caudal subnucleus, which serves as the primary relay for orofacial nociception, and information reaches the thalamus and cortex from there [11].

The key result of this setup is convergence. The poor localization and referral patterns that clinicians experience are often due to the fact that information from more than one peripheral territory is received by the second-order neurons of the trigeminal brainstem complex. This convergence not only underlies the clinical and mechanistic similarity between endodontic pain and TMD, but also accounts for the ability of an insult to occur in one tooth and trigger symptoms that do not. This convergence also helps to explain the clinical and mechanistic overlap between endodontic pain and TMD, as well as the fact that an insult can occur in one tooth and lead to symptoms outside of that tooth [12].

Peripheral sensitization in endodontic pain

Peripheral sensitization begins with the inflammation in the pulp. Inflammatory mediators reduce the activation threshold of nociceptors, increase the recruitment of previously silent fibres, and enhance the response of the trigeminal afferents to further stimulation. This peripheral sensitization is a mechanism that can be correlated with hyperalgesia and allodynia, which are common parts of pulpitis and apical periodontitis. Molecular studies of trigeminal neurons following injury have revealed changes in expression of ion channels and receptors that make these neurons more excitable and maintain the afferent barrage, which is now known as the initial step in more persistent states of pain [13].

Notably, the trigeminal ganglion seems to react in a different manner to peripheral nerve injury as compared to the dorsal root ganglion. Comparative transcriptomic studies have indicated that there are region-specific molecular programmes in the trigeminal ganglion compared with the dorsal root ganglion following equal peripheral nerve injury, which might underlie the face's unique susceptibility to chronic pain following injury [14]. These conclusions provide some insight into why such periapical or periapical inflammation, as well as instrumentation or over-instrumentation, extrusion of materials, or periapical inflammation, may have disproportionate or prolonged sensory effects when the intervention is conducted on the tooth.

Peripheral sensitization in endodontic pain

In cases of intense, repetitive, or prolonged peripheral nociceptive input, peripheral input can intensify central nervous system (CNS) processing and lead to central sensitization: a central amplification of the processing of peripheral input that results in an amplification of peripheral input that is not related to the intensity of the peripheral drive. Central sensitization in the trigeminal system involves increased excitability of neurons in the subnucleus caudalis, increased receptive fields, and decreased descending inhibition. The same process that seems to be involved in the chronification of TMD pain, in which changed brain functioning and central sensitization of pain-producing circuits play a central role, is implicated here in the peripheral-to-central transition [15]. Mechanistic literature is increasingly considering central sensitization as the key process in which a localized, nociceptive issue turns into a pervasive and self-sustaining issue. In this paradigm, insults to the endodontic system are not just localized but can also trigger central circuits that modulate masticatory muscle and joint nociception, if enough afferent barrage is produced. This would suggest that the pulpal/periapical lesion might be involved in the development, exacerbation, or perpetuation of TMD-type pain and that the lesion itself could serve to promote central sensitization after it has occurred [16].

Neuroplasticity and brain-level changes

Chronic orofacial pain has direct evidence from neuroimaging that it alters brain structure. In TMD, structural and functional magnetic resonance imaging (MRI) studies reveal changes in gray matter, cerebral blood flow, and functional connectivity of areas involved in sensory-discriminative and affective aspects of pain, along the trigemino-thalamo-cortical pathway [17]. These results place TMD not only as a joint or muscle disease, but one that has a definite central signature.

Since both endodontic and TMD nociception travel ascendingly by the same pathway, it is logical that the same plastic processes may be involved with persistent pulpal or post-endodontic pain. To characterise this central involvement and differentiate peripheral from centrally maintained orofacial pain, neurophysiological assessment of the trigeminal nociceptive system (electrophysiological and surface electromyographic techniques) has been suggested [18]. Finally, quantitative sensory and psychophysical testing of the TMJ and jaw muscles can give the clinician a window into the mechanisms of sensitization, in addition to the information that can be gained from pain history [19].

The most remarkable relationship between endodontic treatment and chronic trigeminal pain is post-traumatic trigeminal neuropathy (PTTN). An injury to trigeminal nerve fibers, either as a result of the inflammatory process, instrumentation, extrusion of irrigants or filling materials, or proximity between the root apex and the inferior alveolar nerve, can create a neuropathic pain state that persists after the initial disease is treated. A systematic review of endodontic therapy identified that PTTN is more commonly seen in the mid-40s female population, and it can occur from three to 48 months after root canal treatment, with a majority of cases occurring during the first 10 years after treatment, and often presenting with confusing symptoms and unnecessary re-treatment [20].

From endodontic insult to post-traumatic trigeminal neuropathy

PTTN is a complex disorder with a peripheral pathophysiology and central pathophysiology, which involve ectopic discharge, peripheral and central sensitization, phenotypic switching of injured neurons, and genetic predisposition [8,13]. Recent reviews and research on PTTN neurobiology have focused on the molecular changes that follow nerve lesions in the trigeminal ganglion, which contribute to chronic pain after surgery and may account for the fact that a subpopulation of patients develop neuropathic pain after surgery [21]. This continuum from acute pulpal nociception, peripheral sensitization, central sensitization, and, in susceptible patients, frank neuropathy begins a routine endodontic treatment and leads to a chronic pain disorder clinically indistinguishable from or, often accompanied by, TMD.

Clinical convergence and comorbidity and diagnostic pitfalls

In reality, the neurobiological commonalities come to light as diagnostic confusion. Dentoalveolar pain and other idiopathic orofacial pain conditions may be frequently associated with TMD and headache and may be present in the same patient at the same time [22]. Tooth pain alone is not sufficient to confirm the mechanism; therefore, tooth-related pain can be a result of classic odontogenic disease, a sensitized structurally normal tooth, referred muscle pain, or the early stages of a neuropathy evolving. A recent tertiary-center study of patients referred with a trigeminal pain diagnosis revealed that a significant proportion of patients had either alveolar or post-dental neuropathic pain, and also that intraoral changes in sensation, onset after dental intervention, and pain despite dental intervention were the most helpful discriminatory features. Some dental procedures were specifically mentioned as being a potential source of these inferior alveolar or related nerve injuries, such as endodontics [23].

There is a particular kind of risk when this convergence occurs: iatrogenic escalation. If pain is incorrectly localized to a tooth or joint, patients may receive repeated irreversible treatment, such as re-treatment, apicectomy, extraction, or bold occlusal manipulation, without a specific, centrally mediated or neuropathic cause, and without any improvement. This concern has been explicitly stated in the TMD literature, as a disease of patient vulnerability and iatrogenesis, in which the well-intentioned but mechanism-ignorant treatment keeps the disease alive, instead of healing it [24]. As with the endodontic-TMD junctional area, it is important to be able to determine when pain is centrally mediated, to prevent damage.

Therapeutic implications

If endodontic pain and TMD are considered to be two expressions of the same problem, then their care and management will change. If central sensitization or neuropathy is suspected, treatment may not be successful if the peripheral target is the only area to be treated and may even worsen symptoms. Established post-traumatic trigeminal neuropathic pain therefore focuses on pharmacologic and, in some cases, surgical approaches to sensitised peripheral and central pathways with a high emphasis on early and accurate diagnosis as this is a key determinant of outcome [25]. Topical and locally applied agents targeting the intraoral environment are one method for treating peripheral aspects of neuropathic orofacial pain [26], and new preclinical research is ongoing to investigate additional methods of antinociception as adjuncts [27,28]. In all of these approaches, the key is when. Prognosis becomes more dire with continued pain and central changes, which occur during the early window. This suggests increased clinical awareness: If post-endodontic pain does not resolve as expected, if there is sensory disturbance with the pain, or if the pain is present with pain in the masticatory muscles and joints, then the clinician should look for a sensitized or neuropathic pain mechanism before resorting to more peripheral treatment [29,30].

Summary of key studies

Table 1 highlights five representative studies from across the review period (2020-2026) - one anchoring each of the central themes: brain-level neuroplasticity in TMD, the endodontic origin of trigeminal neuropathy, the neurobiology of post-traumatic trigeminal neuropathy, neurophysiological assessment, and clinical diagnostic overlap. Reference numbers correspond to the full reference list [31-34].

**Table 1:** Five representative studies spanning the review period (2020–2026)

Ref.	Author(s), year	Article type	Focus	Key relevance to the endodontic pain – TMD link
[11]	Yin et al., 2020	Systematic review	Structural and functional MRI in TMD pain	Demonstrates central neuroplastic changes involving the trigemino-thalamo-cortical and other pain-processing networks in TMD.
[8]	Al-Khudhairy et al., 2022	Systematic review	Post-traumatic trigeminal neuropathy after endodontic therapy	Documents PTTN following endodontic treatment and summarizes its clinical characteristics, demographic profile, and onset following treatment
[28]	Eliav et al., 2024	Review	Neurobiology and pathophysiology of PTTN	Describes peripheral and central mechanisms, neuronal changes, and genetic factors involved in the development and persistence of PTTN
[34]	Raciti et al., 2025	Literature review	Neurophysiological examination in orofacial pain and TMD	Supports the use of neurophysiological assessment to characterize orofacial pain mechanisms and distinguish peripheral from centrally maintained pain
[23]	Shemesh et al., 2026	Retrospective study	Post-dental/alveolar trigeminal pain versus trigeminal neuralgia	Highlights diagnostic misclassification at the dental–trigeminal interface and the importance of recognizing post-dental and alveolar nerve-related pain

Limitations

There are a number of caveats that ratify these conclusions. Most of the mechanistic evidence for the correlation between the endodontic insult and central sensitization comes from animal studies of trigeminal nerve injury and studies of trigeminal neuroimaging in TMD patients, but not from a longitudinal study of endodontic patients progressing to chronic pain. There is a lack of understanding about persistent post-endodontic pain and TMD and a great deal of variability in the diagnosis from each study; thus, the synthesis is difficult. Further research on prospective cohorts of patients with psychophysical, neurophysiological, and imaging tests after endodontic treatment would assist in understanding what patients become sensitised or neuropathic and why. The identification of the molecular program in the trigeminal system that is different from the spinal system will hopefully lead to targeted preventive and therapeutic interventions.

## Conclusions

There are a number of caveats that ratify these conclusions. Most of the mechanistic evidence for the correlation between the endodontic insult and central sensitization comes from animal studies of trigeminal nerve injury and studies of trigeminal neuroimaging in TMD patients, but not from a longitudinal study of endodontic patients progressing to chronic pain. There is a lack of understanding about persistent post-endodontic pain and TMD and a great deal of variability in the diagnosis from each study; thus, the synthesis is difficult. Further research on prospective cohorts of patients with psychophysical and neurophysiology and imaging tests after endodontic treatment would assist in understanding what patients become sensitised or neuropathic and why. The identification of the molecular program in the trigeminal system that is different from the spinal system will hopefully lead to targeted preventive and therapeutic interventions.
